# The Phase‐Amplitude Coupling Changes Induced by Smoking Cue After 12‐H Abstinence in Young Smokers

**DOI:** 10.1111/adb.70048

**Published:** 2025-05-19

**Authors:** Zhiwei Ren, Juan Wang, Yongxin Cheng, Yuxin Ma, Youwei Dong, Yiming Lu, Ting Xue, Gengdi Huang, Dahua Yu, Fang Dong, Kai Yuan

**Affiliations:** ^1^ School of Digital and Intelligent Industry Inner Mongolia University of Science and Technology Baotou Inner Mongolia China; ^2^ Department of Addiction Medicine, Shenzhen Kangning Hospital Shenzhen Mental Health Center Shenzhen China; ^3^ State Key Laboratory of Chemical Oncogenomics, Guangdong Provincial Key Laboratory of Chemical Genomics Peking University Shenzhen Graduate School Shenzhen China; ^4^ School of Automation and Electrical Engineering Inner Mongolia University of Science and Technology Baotou Inner Mongolia China; ^5^ Life Sciences Research Center, School of Life Science and Technology Xidian University Xi'an Shaanxi China; ^6^ Ganzhou City Key Laboratory of Mental Health The Third People's Hospital of Ganzhou City Ganzhou Jiangxi China; ^7^ Engineering Research Center of Molecular and Neuro Imaging Ministry of Education Xi'an Shaanxi China; ^8^ Xi'an Key Laboratory of Intelligent Sensing and Regulation of Trans‐Scale Life Information, School of Life Science and Technology Xidian University Xi'an Shaanxi China

**Keywords:** craving, electroencephalography, functional connectivity, phase–amplitude coupling, young smokers

## Abstract

Tobacco use causes more than 8 million deaths globally each year, and the number of younger smokers is growing. It is of great practical importance to explore the underlying neural mechanisms behind the behaviour of young smokers. During cue‐induced craving, reward system in the brain generates neural oscillations at specific frequencies. The phase–amplitude coupling (PAC) can capture interactions between these frequencies and may be a more sensitive quantitative indicator for characterizing abnormal neural oscillations in smokers. We monitored the electroencephalography (EEG) data of 30 young smokers during a cue task after 12 h of abstinence, dividing the data into the neutral and smoking‐related groups based on different experimental stimuli to analyse the relationship between PAC and craving. In addition, we computed the functional connectivity (FC) under the PAC mechanism. The results showed that the young smokers exposed to smoking‐related cues under short‐term abstinence conditions had significantly lower PAC values and reduced FC strength in the right prefrontal cortex. In contrast, there was a significant increase in PAC values in the parietal cortex and enhanced FC strength. The correlation analysis showed significant correlations between PAC values and craving. These findings demonstrate for the first time that PAC abnormalities in young smokers exposed to smoking‐related cues under short‐term abstinence conditions may be related to craving and inhibitory control.

## Graphical abstract

Whole‐brain PAC and functional connectivity analyses in smoking quitters using the Cue‐Task revealed that smoking‐related stimuli decreased prefrontal PAC and connectivity, while increasing PAC and enhancing connectivity in the posterior parietal cortex. PAC may be a sensitive indicator of craving representations.

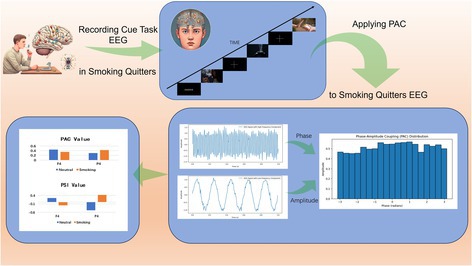

## 1 Introduction

Tobacco use represents a significant risk factor for global public health. Recent data released by the World Health Organization (WHO) indicate that the worldwide prevalence of smoking has surpassed 1.2 billion individuals, constituting approximately 15% of the global population [1]. More than 6 million people worldwide die of smoking‐related diseases every year [2]. Recent studies have demonstrated a correlation between early initiation of smoking and the likelihood of developing into lifelong smokers [3, 4, 5]. Relying on substances such as nicotine can result in neuroplastic alterations within brain circuits, particularly those involved in the processing of reward [6, 7], and may also lead to impairments in cognitive function and inhibitory control [8, 9]. Previous electroencephalography (EEG) studies have primarily used spectral analysis to characterize abnormal neural oscillations in smokers [10]. However, some analyses of the EEG power spectra of smokers' brains have failed to observe significant abnormal neural oscillations caused by smoking. Therefore, a more reliable and sensitive indicator is needed to quantify the abnormal neural oscillations in the brain of smokers [11].

Neural oscillation refers to the repetitive activity of neurons within the central nervous system [12]. It is characterized by distinct frequency‐dependent coupling properties [13]. Cross‐frequency coupling (CFC) is a type of EEG electrical signal based on electrophysiological technology, which reflects the statistical relationship between the amplitude and phase combinations across different frequency bands in the brain [14, 15]. Phase–amplitude coupling (PAC) is the result of mutual coordination of EEG activity between different frequency bands and plays an important role in processes such as attention, memory, perception and motor control [16, 17]. PAC can constitute a flexible mechanism for combining information across different time scales within a local cortical network [18, 19]. For example, during the process of memory formation in the brain, there is a noticeable coupling phenomenon between EEG signals in the low alpha band and high gamma band that are collected by the hippocampus [20]. This cross‐frequency coupling facilitates the transfer of information among neurons, particularly by enhancing synchronization between low frequency signals at specific phases and high frequency signals. It has been established that the PAC of electrical signals generated within the brain reflects communication and information encoding between neurons, at both microscopic and macroscopic scales of functional brain regions [21, 22].

Smoking cue reactivity is an important feature of nicotine addiction [23]. This activity involves several EEG features, such as P300 and slow positive waves in the time domain and alpha oscillation features in the frequency domain [24]. Individuals with various types of addictions exhibit distinct behavioural patterns following withdrawal [25, 26]. Craving increases when smokers are exposed to smoking‐related cues after short‐term abstinence [27] and is predicted to increase delta activity and theta‐band power [28]. The brain regions responsible for inhibitory control exert a certain degree of inhibition on the reactivity to smoking cues [29, 30]. Given the changes in multiple frequency bands such as δ, θ, α, β, and γ in the brain induced by smoking cue reactivity, there may be alterations in PAC within the smoking‐related brain regions. Thus, in a way, PAC may represent the ability to inhibit control [31], and at the same time, PAC may be associated with representations of smoking cravings. Although few studies have explored the mechanism of PAC under smoking cue reactivity‐induced craving, there has been some research to support that PAC can be used as an important simultaneous measure and holds promise as a marker of abnormal neural oscillations in the brain of smokers [32]. Additionally, functional connectivity (FC) analysis provides valuable insights into the brain's network dynamics during cognitive tasks and the processing of external stimuli [33], offering critical scientific evidence for understanding neurological disorders, cognitive functions and addictive behaviours [34, 35]. Therefore, in the present study, we examined the mechanism of CFC in young smokers under short‐term abstinence. Given that abnormalities in CFC may alter FC by affecting synchronized activity in different regions of the brain, connectivity analyses were also performed to clarify the mechanism of CFC.

Based on the above summary, we recorded cue task‐state EEG data from 30 young smokers after 12 h of abstinence to investigate the neural mechanisms underlying nicotine addiction. Using PAC and FC metrics, we explored the following hypotheses: (1) Under short‐term abstinence conditions, smoking cue exposure may cause significant changes in PAC in young smokers. (2) PAC may be related to representations of inhibitory control. (3) PAC may be a novel indicator of craving representation. (4) Smoking cue exposure may cause significant changes in the strength of FC in young smokers under short‐term abstinence conditions.

## 2 Materials and Methods

### 2.1 Subject Screening

Thirty young male smokers, all Han Chinese and right‐handed, were recruited from Inner Mongolia University of Science and Technology through smoking‐related advertisements. Due to the low number of female smokers, only males were included in the study. The participants had a mean age of 20.7 ± 0.9 years. The inclusion criteria were as follows: (1) the Fagerstrom Test for Nicotine Dependence (FTND) score ≥ 2 points [36]; (2) nicotine dependence consistent with the criteria for tobacco use disorder as outlined in the Diagnostic and Statistical Manual of Mental Disorders (DSM); (3) carbon monoxide level not lower than 7 ppm (parts per million) measured before the experiment; (4) abstinence from smoking for less than three months in recent years; and (5) a smoking history of at least 10 cigarettes per day in the recent past.

The exclusion criteria were as follows: (1) dependence on alcohol and coffee, past medical history, taking medication affecting cognitive function, left‐handedness and spatial phobic; and (2) psychiatric, neurological or epileptic disorders. Demographic characteristics of the subjects are presented in Table 1. Since the EEG data were divided based on smoking and neutral cue conditions during the cue‐task, the two data groups involved the same subjects, ensuring no significant differences in characteristics such as age, height, weight or years of education. The Smoking Cravings Scale was used to assess the subjects' craving levels, with responses ranging from 1 (strongly disagree) to 7 (strongly agree) [37]. To minimize the acute effects of nicotine, participants were asked to abstain from smoking for 12 h before the experiment, based on previous studies indicating that even a single 21 mg dose of transdermal nicotine can affect EEG activation [38, 39]. The subjects were instructed to cleanse their scalps to ensure acquisition of high‐quality EEG data. Upon completion of the experiment, the subjects received monetary compensation for their participation.

**TABLE 1:** Demographic characteristics of participants (mean ± SD).

Demographic variables	Young smokers (*N* = 30)
Sex (male), *N* (%)	30 (100%)
Age range (years)	19–22
Age (years), mean ± SD	20.7 ± 0.9
Education years (years), mean ± SD	14.03 ± 0.48
Age at smoking onset, mean ± SD	15.23 ± 2.77
FTND score, mean ± SD	4.73 ± 1.59
Packyears, mean ± SD	2.92 ± 2.26
Cigarettes per day (CPD), mean ± SD	14.03 ± 4.36
Duration of smoking, mean ± SD	4.33 ± 2.17

### 2.2 Experimental Design and Procedures

**FIGURE 1:**
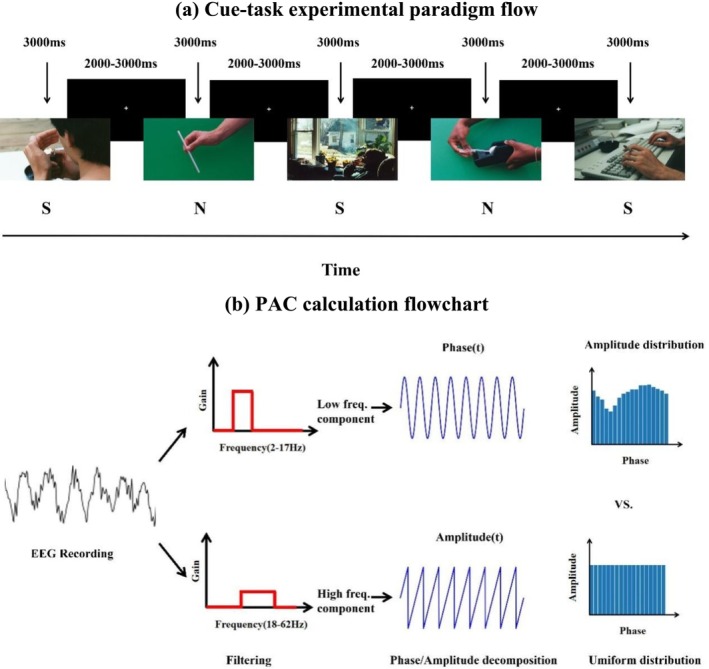
Experimental design and computational procedure for PAC (PAC: phase–amplitude coupling). (a) Cue‐task experimental paradigm flow (S: Smoking cue reactivity N: Neutral cue reactivity), (b) PAC calculation flowchart. The flowchart shows the PAC calculation process in detail.

The experimental paradigm was developed using E‐Prime 2.0 software (Psychology Tools, Pittsburgh, PA, USA). The experiment included 50 smoking‐related images and 50 neutral images, all sourced from the International Smoking Picture Library. The smoking‐related images depicted scenes such as smoking, lighting cigarettes and burning cigarettes, while the neutral images featured scenes such as landscapes, straws and offices. The two sets of images were matched in all aspects except for smoking‐related content. Each image was presented for approximately 3000 ms, and the presentation order was randomized. A random interstimulus interval of 2000–3000 ms was allowed between the end of one image and the start of the next one (Figure 1a).

The participants were instructed to abstain from smoking for 12 h before the experiment and to avoid late nights and insomnia. Prior to the experiment, each subject underwent a carbon monoxide concentration test and completed a pre‐experimental craving scale. A 64‐lead electrode cap was placed on the subject's head, and conductive paste was applied to ensure that the electrode impedance remained below 10 kΩ. Once all preparations were complete, the experiment began and lasted for approximately 10 min. After the experiment, the participants filled out a post‐experiment craving scale, and the EEG cap was then removed.

### 2.3 Data Acquisition and Pre‐Processing

EEG data were collected using a 64‐channel signal recorder from Brain Products, Germany, with silver chloride electrodes embedded in magnetically harmonized caps, arranged according to the international 10–20 system. FCz served as the online reference electrode. The sampling rate was set at 1000 Hz, and the impedance between the electrodes and scalp was maintained below 10 kΩ to ensure high‐quality data. All data were recorded online using Brain Products' Recorder software.

The obtained EEG data were analysed offline using MATLAB R2021b, following these steps: (1) band‐pass filtering: a high‐pass filter at 0.1 Hz and a low‐pass filter at 100 Hz were used to remove nonphysiological signals; (2) notch filtering: 50 Hz and 100 Hz notch filters were used to eliminate industrial frequency interference; (3) electrode re‐referencing: to minimize EEG signal interference from electrocardiogram (ECG) and hemispheric potential differences, the average potentials of the bilateral mastoids (TP9 and TP10) were used as the new reference, and FCz was reclassified as a recording electrode; (4) interpolation of bad leads: electrodes that failed to capture accurate EEG signals, referred to as bad leads, were removed and replaced with neighbouring electrodes to improve data quality; (5) rejection of bad segments: segments containing artefacts from actions such as swallowing saliva and body shaking were discarded to facilitate cleaner analysis; (6) independent component analysis (ICA): ICA was performed to remove artefacts from eye movements and blinks, preserving as much of the true signal as possible.

### 2.4 PAC Calculation

The obtained EEG signals were divided into two frequency bands based on the frequency ranges of interest and filtered separately (Figure 1b). Specifically, we selected a combination of phase (2–16 Hz) and amplitude (18–62 Hz) ranges to investigate the coupling between low‐frequency phases (δ, θ, α and β) and high‐frequency $\left(\gamma \right)$ amplitude within each electrode. The Hilbert transform was applied to extract the phase of the low frequency and the amplitude of the high frequency. Specifically, we employed the modulation index (MI) method [40] to quantify PAC, and cluster‐based permutation test was applied to identify significant electrodes and frequency bands showing differences in PAC between the two groups. To improve the sensitivity and robustness of the measurements and to reduce parasitic coupling [41], we normalized PAC. Specifically, we computed PAC value every 3 s, averaged the PAC values over this period of time and used this average as the final PAC value. The computation of PAC was done using the TensorPac toolbox [42].

### 2.5 FC Calculation

Phase slope index (PSI) within frequency: To assess remote connectivity of signals in the source space during the smoking craving task, we computed PSI between different electrodes. PSI is a robust metric for estimating information flow between two signals [43]. It quantifies directionality by inferring whether one signal is leading or lagging relative to another based on the slope of the phase difference in a predefined frequency domain [44]. PSI assumes that a constant lag in the time domain can be transformed into a linearly varying phase difference across frequency. To minimize edge artefacts, we used a relatively long 3000‐ms segment, after applying a Hanning window and extracting the Fourier coefficients. PSI values were calculated for the smoking stimulation condition and the neutral condition at the F4 electrode versus the other 61 electrodes in the frequency ranges of 6–8 Hz, and 56–62 Hz. Similarly, we computed PSI values at the P4 electrode against the other 61 electrodes in the 12–15 Hz and 30–40 Hz ranges. Significant positive PSI values indicated the information flow from the F4 and P4 electrodes to the other electrodes, while negative values suggested the flow of information in the opposite direction. Values close to zero indicated no significant information flow.

### 2.6 Statistical Analysis

Results were analysed using paired *t*‐tests and independent‐samples *t*‐tests. Cluster‐based permutation tests in MNE‐Python were conducted to correct for multiple comparisons and to identify statistical differences in PAC, with significance set at *p* < 0.05 [45]. The linear relationship between PAC values and craving scores was assessed using the Pearson correlation coefficient. For within‐frequency PSI analyses, we averaged the PSI values across electrode pairs and employed paired *t*‐tests to evaluate directional differences between the neutral and smoking groups.

## 3 Results

### 3.1 Abnormal Changes in PAC

**FIGURE 2:**
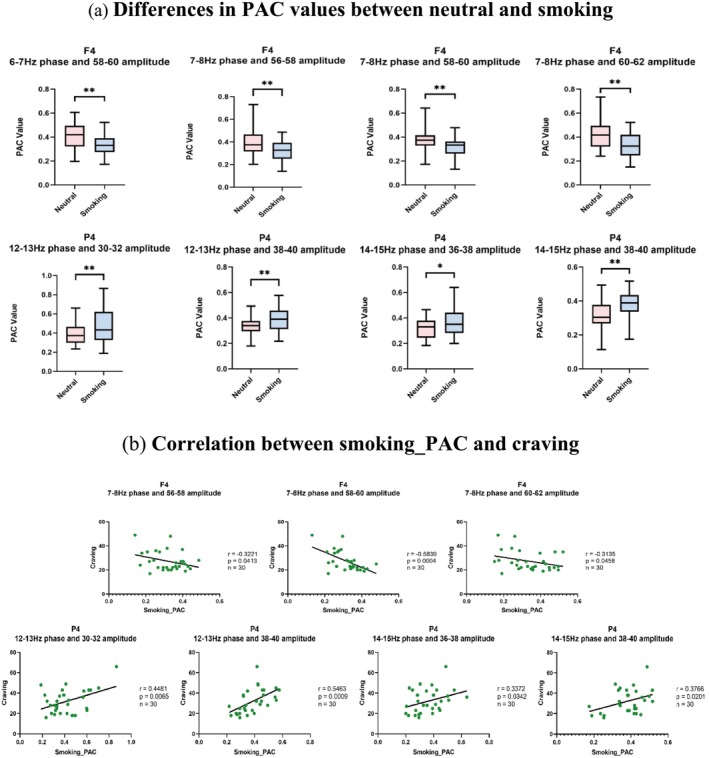
Differences in PAC in young smokers after smoking cue reactivity. (a) Differences in PAC values between neutral and smoking (ns, not significant; *, *p* < 0.05; **, *p* < 0.01). (b) Correlation between smoking_PAC and craving.

For young smokers with short‐term abstinence, the PAC values at the F4 electrode showed significant differences between the smoking stimulus and neutral pictures. The clusters that were significantly different between the two groups were averaged and a paired‐sample *t*‐test was performed. The results showed significant reductions in phase frequency at 6–8 Hz and amplitude frequency at 56–62 Hz (Figure 2a). Frequency specificity of PAC deficits at the F4 electrode in young smokers with short‐term abstinence in smoking stimulation experiments was demonstrated. It is noteworthy that the electrodes with the significantly improved PAC values were distributed in the right prefrontal cortex. Similarly, the PAC values at the P4 electrode showed significant increases in phase frequency at 12–15 Hz and amplitude frequency at 30–40 Hz during the smoking stimulus (Figure 2a). This frequency‐specific increase in PAC was observed in the parietal cortex of young smokers in the experiment.

Correlation analysis revealed that the PAC values at the F4 electrode for phase frequency of 7–8 Hz and amplitude frequency of 56–62 Hz showed a significant negative correlation with craving scores (Figure 2b). In contrast, the PAC values for phase frequencies of 12–15 Hz and amplitude frequencies of 30–40 Hz at the P4 electrode showed a significant positive correlation with the craving values (Figure 2b).

To further explore the mechanism of PAC, FC was computed on the basis of the PAC mechanism.

### 3.2 Abnormal Changes in FC

**FIGURE 3:**
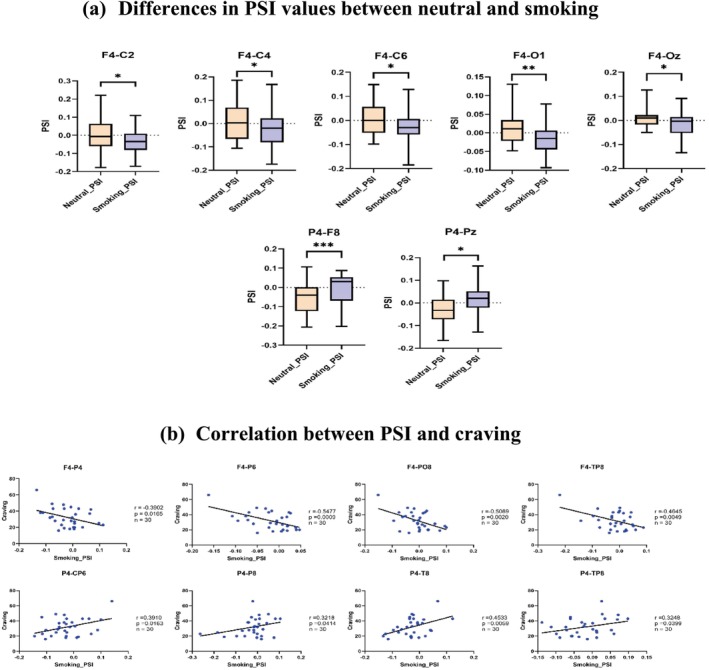
Differences in PSI in young smokers after smoking cue reactivity. (a) Differences in PSI values between neutral and smoking (ns, not significant; *, *p* < 0.05; **, *p* < 0.01). (b) Correlation between PSI and craving.

When young smokers with short‐term abstinence viewed neutral images, the PSI values between the F4 electrode and C2, C4, C6, O1 and Oz electrodes were close to zero, indicating minimal information exchange between F4 and these electrodes. However, when viewing smoking‐related images, the PSI values between F4 and C2, C4, C6, O1 and Oz significantly increased, showing information flow from these electrodes to F4 (Figure 3a). Similarly, for the P4 electrode, when viewing neutral images, the PSI values with F8 and Pz electrodes were negative, indicating information flow from F8 and Pz to P4. When exposed to smoking‐related images, the PSI values became positive, indicating a reversal of information flow, from P4 to F8 and Pz (Figure 3a).

Correlation analysis of the PSI values showed that the electrode pairs F4–P4, F4–P6, F4–PO8 and F4–TP8 had significant negative correlations with craving scores (Figure 3b). Conversely, the PSI values for the electrode pairs P4–CP6, P4–P8, P4–T8 and P4–TP8 showed significant positive correlations with craving (Figure 3b).

## 4 Discussion

This study is the first to investigate the relationship between changes in PAC and craving in young smokers with short‐term abstinence during smoking cue exposure. We found that smoking cue exposure caused a decoupling of phase frequencies (7–8 Hz) and amplitude frequencies (56–62 Hz) in the right prefrontal cortex compared with neutral stimulation (Figure 2a). Additionally, FC between the F4 electrode and other electrodes was significantly reduced (Figure 3a), and both smoking_PAC and PSI showed significant negative correlations with craving (Figure 2b and Figure 3b). These findings suggest that decoupling in the right prefrontal cortex may be related to deficits in inhibitory control in young smokers. In contrast, smoking stimulus images increased the coupling of phase frequencies (12–15 Hz) and amplitude frequencies (30–40 Hz) in the parietal cortex (Figure 2a). FC between the P4 electrode and other electrodes was also enhanced (Figure 3a). Correlation analyses revealed significant positive associations of smoking_PAC and PSI with craving (Figure 2b and Figure 3b), indicating that increased PAC in the parietal cortex may be linked to heightened craving representation.

Recent studies have shown that PAC can be used as a new indicator to help us understand and study the synchronization of neural oscillations at different frequencies within the brain, and PAC can support dynamic communication within the brain [46, 47]. PAC has attracted much attention in the study of neurological disorders [48], such as Alzheimer dementia, bipolar disorder, attention deficit hyperactivity disorder, autism spectrum disorder, obsessive‐compulsive disorder, depression, social anxiety disorder and schizophrenia [49]. Brinkmeyer et al. highlighted the critical role of the prefrontal cortex in sensory gating, proposing that deficits in cortical inhibition among nicotine‐dependent smokers underlie impairments in P50 gating and related abnormalities, such as high‐frequency oscillations in frontal brain regions [50]. Notably, these deficits were found to be more pronounced in heavy smokers. Our findings align with and extend this perspective by demonstrating that exposure to smoking cues elicits distinct patterns of PAC and FC in both the prefrontal and parietal cortices. These neural dynamics are closely associated with heightened craving intensity and deficits in inhibitory control, further supporting the notion that smoking‐related neural adaptations involve disruptions in cortical inhibition and functional network organization.

Previous findings have suggested that smoking impacts inhibitory control in young smokers [51]. Kirmizi‐Aslan et al. demonstrated that inhibitory control is correlated with theta frequency in the frontal cortex, and that a decrease in theta frequency decreased inhibitory control [52, 53]. Similarly, James reported found that theta rhythm waves in the prefrontal cortex appeared to reflect the co‐computation used to achieve inhibitory control [54]. Zhang Peng et al. demonstrated that reduced theta rhythms induced weaker theta–gamma PAC in the right prefrontal cortex during the Go/NoGo task [31]. Our findings align with this, as smoking stimuli induced a reduction in PAC values in the prefrontal cortex at a phase frequency of 7–8 Hz and an amplitude frequency of 56–62 Hz compared with neutral images. This suggests that self‐regulating mechanisms of neural activity are disrupted, leading to reduced cortical activation and phase–amplitude decoupling. Our findings show that the theta–gamma PAC in the right prefrontal cortex may be associated with prefrontal inhibitory control and may be a new indicator for characterizing inhibitory control. This result aligns with the report of Zhang Peng et al. Additionally, the parietal cortex—which plays a critical role in visual processing [55] and attentional focus [56]—has been shown in prior research to be more responsive to smoking stimuli in smokers than to neutral conditions [57], with its involvement in craving representation [58]. Our study showed that smoking stimuli led to increased coupling of phase frequencies (12–15 Hz) and amplitude frequencies (30–40 Hz) in the parietal cortex of young smokers with short‐term abstinence. This may indicate that smoking stimuli heightened attention in these individuals, leading to an over‐allocation of cognitive resources to smoking cues, thereby intensifying craving representations in the parietal cortex. This is consistent with previous findings that the β band is associated with arousal, attention and vigilance, and that activation of the β band due to smoking stimulus conditioning reflects an increased allocation of resources to the smoking stimulus, i.e., a processing bias [53]. Thus, we think that an increase in the coupling of phase frequencies of 12–15 Hz and amplitude frequencies of 30–40 Hz in the parietal cortex may be related to the characterization of craving.

We further examined the relationship between PAC values and craving levels, and found through Pearson correlation analysis that the PAC values at the F4 electrode exhibited a significant negative correlation with craving (Figure 2b). Specifically, higher craving levels in short‐term abstinent smokers were associated with lower PAC values. This suggests that increased craving, likely due to impaired inhibitory control in the right prefrontal cortex, is accompanied by reduced theta‐band (7–8 Hz) activity, which leads to decreased coupling between the phase frequency of 7–8 Hz and the amplitude frequency of 56–62 Hz. In contrast, the PAC values in the parietal cortex showed a significant positive correlation with craving, indicating that heightened craving enhanced beta‐band (30–40 Hz) activity. This also resulted in increased coupling of the phase frequency of 12–15 Hz and the amplitude frequency of 30–40 Hz in the parietal cortex. To further validate the PAC‐related conclusions obtained in this study, PSI was used to perform the connectivity analysis in the mechanism of CFC. We discovered an interesting neuronal pattern that revealed the directionality of information flow in the time domain. Specifically, our results showed that although the smoking stimulation condition caused an increase in the strength of FC between the prefrontal cortex F4 electrode and the other electrodes in young smokers with short‐term abstinence, the flow of information was from the other electrodes to the F4 electrode (Figure 3a). We assume that the main reason for this phenomenon may be due to the occurrence of the right prefrontal cortex decoupling, where the central and occipital cortex worked together with the prefrontal cortex to accomplish decoupling. This aligns with findings from Palermo et al. [59], who demonstrated the critical role of the prefrontal cortex in behavioural control using the Go/NoGo task. Their work highlighted how declining frontal network function is associated with control deficits during task performance, consistent with the reduction in FC seen in our results. In contrast to the F4 electrode, the PSI values at the P4 electrode significantly increased under the smoking stimulation condition, with information flowing from the P4 electrode to the other electrodes (Figure 3a). We suggest that this pattern may be due to heightened parietal cortex activation, leading to increased PAC coupling, stronger FC between the parietal and prefrontal regions and enhanced craving representations during smoking stimuli. These PSI results provide further insight into the neural mechanisms underlying PAC in relation to craving and inhibitory control, illustrating how different cortical regions interact under specific stimulus conditions.

Clustered neurons generate activity patterns in the cerebral cortex, representing task‐related information that can be transmitted across synapses to spatially distributed brain regions [60]. Notably, such task‐related neuronal oscillations are absent in resting EEG signals, emphasizing the unique nature of these responses. In an exploratory study, we used smoking cue reactivity task‐related EEG signals to perform a comprehensive analysis of abnormal neural oscillatory coupling activity between low‐frequency phases and high‐frequency amplitudes within the brain of young smokers in short‐term abstinence, raising the possibility that cross‐frequency PAC can serve as a surrogate marker for smoking problems. Thus, our study provides additional evidence to further understand the neural mechanisms of adolescent smoking addiction. Future studies could combine multiple approaches to explore adolescent smoking addiction.

## 5 Limitations

There are several limitations to this study. First, since the number of female smokers among college students is small, this experiment only focused on male smokers, and the number of samples in this experiment was relatively small. Second, we did not conduct the source analyses. Finally, our experimental design was cross‐sectional, as a result of which we were not able to explore in depth the causal relationship between smoking and EEG signal alterations. Therefore, we will continue to expand our sample size and the scope of our future research, constantly improve our experimental design, and add retrospective analysis. On this basis, we will continue to follow up the changes and effects of nicotine on the plasticity of the brain of young smokers, so as to analyse the problem of young smoking in a more detailed way.

## Author Contributions

Authors declare that Zhiwei Ren, Juan Wang and Kai Yuan designed the studies, with refinements contributed by Dahua Yu. Zhiwei Ren, Yiming Lu, Youwei Dong, Yongxin Cheng, Gengdi Huang and Yuxin Ma performed the research, conducted initial data analysis, created figures and conducted statistical analysis of data. Zhiwei Ren, Fang Dong wrote the major drafts of the paper. Ting Xue, Dahua Yu and Kai Yuan edited sections on the manuscript, All authors have approved the submitted version of the manuscript.

## Ethics Statement

The current study complied with the Declaration of Helsinki and got permission from the Medical Ethical Committee of the First Affiliated Hospital of Baotou Medical College, Inner Mongolia University of Science and Technology. The study was registered in the Chinese Clinical Trial Registry (No. ChiCTR2100042449). Prior to the experiment, each subject signed an informed consent form after fully understanding the procedures and precautions.

## Conflicts of Interest

The authors declare no conflicts of interest.

## Data Availability

The datasets used and analyzed during the current study are available from the corresponding author upon reasonable request.
